# Genetic polymorphisms -137 (rs187238) and -607 (rs1946518) in the interleukin-18 promoter may not be associated with development of hepatocellular carcinoma

**DOI:** 10.1038/srep39404

**Published:** 2016-12-21

**Authors:** Shao-Liang Zhu, Yun Zhao, Xue-Ying Hu, Tao Luo, Zu-Shun Chen, Yu Zhang, Shui-Hua Yang, Lu Zhou, Le-Qun Li

**Affiliations:** 1 Department of Hepatobiliary Surgery, Affiliated Tumor Hospital of Guangxi Medical University, Nanning 530021, China; 2 Department of Chemotherapy, Affiliated Tumor Hospital of Guangxi Medical University, Nanning 530021, China; 3 Department of Radiology, Affiliated Tumor Hospital of Guangxi Medical University, Nanning 530021, China; 4 Department of Ultrasound, Maternal & Child Health Hospital of Guangxi Zhuang Autonomous Region, Nanning 530021, China; 5 Department of Oncology, Nanning First People’s Hospital, Nanning 530021, China

## Abstract

This study meta-analyzed the literature on possible association of polymorphisms -137 (rs187238) and -607 (rs1946518) in the interleukin-18 (IL-18) promoter with risk of hepatocellular carcinoma (HCC). The analysis included 8 case-control studies on the -137 polymorphism (1,318 cases, 2,254 controls) and 7 case-control studies on the -607 polymorphism (1,262 cases, 1,696 controls). None of the five genetic models suggested a significant association between the -137 polymorphism and HCC risk: allelic model, OR 0.99, 95% CI 0.74–1.34, *P* = 0.97; recessive model, OR 0.98, 95% CI 0.65–1.46, *P* = 0.91; dominant model, OR 1.35, 95% CI 0.73–2.52, *P* = 0.34; homozygous model, OR 0.99, 95% CI 0.65–1.49, *P* = 0.95; heterozygous model, OR 0.99, 95% CI 0.66–1.48, *P* = 0.94. Similar results were obtained in subgroup analyses of Asian patients, Chinese patients, or patients with hepatitis B virus (HBV)-related HCC. Similar results were also obtained for the -607 polymorphism across the entire study population as well as in the three subgroups. The available evidence suggests no significant association of the -137 or -607 polymorphisms with risk of HCC in general or specifically of HBV-related HCC. These conclusions should be verified in large, well-designed studies.

Hepatocellular carcinoma (HCC) is one of the most common causes of cancer-related mortality worldwide^1^. Risk of HCC has been clearly linked to chronic infection with hepatitis B virus (HBV) or hepatitis C virus (HCV): these viruses cause chronic hepatitis, liver cirrhosis and liver failure, which increase risk of HCC^2^. Genetic factors are also likely to play a role in HCC, since only a fraction of patients chronically infected with HBV or HCV develop HCC during their lifetime.

Interleukin-18 (IL-18), originally known as interferon-γ (IFN-γ)-inducing factor^3^,^4^,^5^, may participate in HCC pathogenesis. HCC is one of several cancers involving a chronic inflammatory response, and IL-18 is a proinflammatory cytokine that triggers various pathways to protect the host against infection. It promotes a Th1-type immune response^4^, and it activates proinflammatory effector cells that contribute to acute and chronic viral hepatitis as well as induce target cell apoptosis^6^,^7^. IL-18 stimulates cytotoxic T lymphocytes and natural killer cells to produce IFN-γ^8^,^9^,^10^, which helps suppress viral replication and determine the host response to infection^11^.

These findings suggest the possibility that genetic factors affecting the expression or activity of IL-18 may influence risk of HCC. Indeed, numerous case-control studies^12^,^13^,^14^,^15^,^16^,^17^,^18^,^19^ have investigated whether polymorphisms at position -137 (rs187238) and -607 (rs1946518) within the IL-18 promoter influence risk of HCC. Results have been inconclusive and contradictory, prompting us to perform the first comprehensive meta-analysis of all available evidence on these potential associations.

## Results

### Description of studies

**Figure 1:**
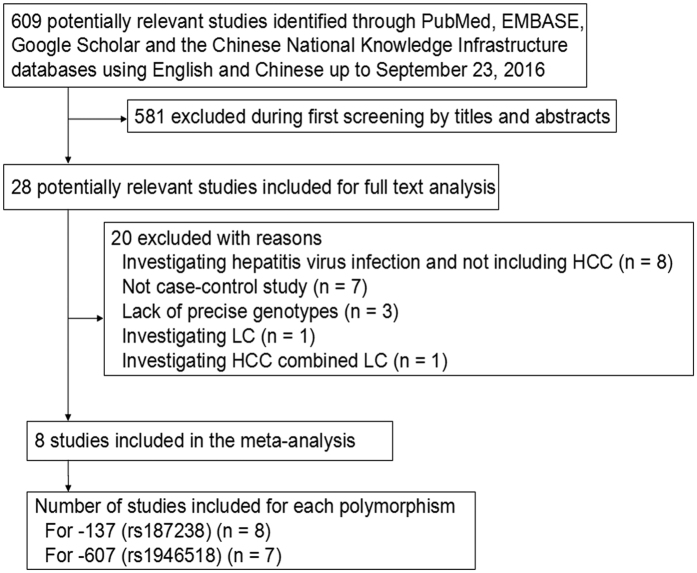
Flowchart of study selection. HCC, hepatocellular carcinoma; LC, liver cirrhosis.

A total of 609 potentially relevant publications published in English or Chinese as recently as September 23, 2016 were systematically identified in PubMed, EMBASE, Google Scholar and CNKI databases (Fig. 1). We excluded 581 studies based on review of the titles and abstracts. We excluded another seven studies^20^,^21^,^22^,^23^,^24^,^25^,^26^ because they were not case-control studies, three studies because they did not report genotypes^27^,^28^,^29^, and eight studies^30^,^31^,^32^,^33^,^34^,^35^,^36^,^37^ because they did not involve patients with HCC. A further two studies^38^,^39^ were excluded because they included either patients with only liver cirrhosis or an aggregated patient population with HCC or liver cirrhosis. In the end, 8 studies^12^,^13^,^14^,^15^,^16^,^17^,^18^,^19^ were included in the final meta-analysis based on our search strategy and inclusion criteria (Table 1). Of these 8 studies, all^12^,^13^,^14^,^15^,^16^,^17^,^18^,^19^ evaluated the association between the -137 polymorphism and HCC risk (1,318 cases, 2,254 controls), while 7 studies^12^,^14^,^15^,^16^,^17^,^18^,^19^ evaluated the association between the -607 polymorphism and HCC risk (1,262 cases, 1,696 controls). The distribution of genotypes in controls was consistent with Hardy-Weinberg equilibrium (HWE, *P* > 0.05) in all but two studies^13^,^15^. The overall quality of the included studies was adequate. The mean score for the included studies was 6.25 (Table 2).

### Meta-analysis of data on the -137 (rs187238) polymorphism

**Figure 2:**
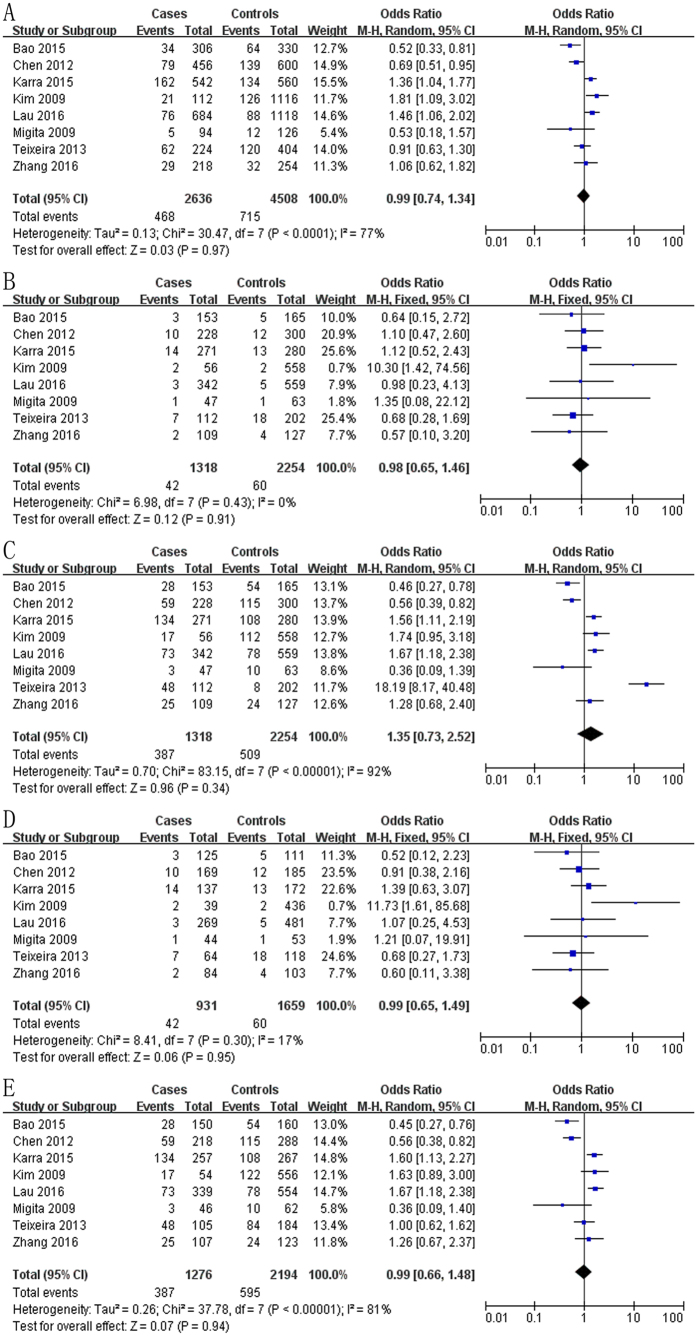
Forest plot describing the association between the -137 polymorphism (rs187238) and risk of across all study participants according to different genetic models. (**A**) allelic (C-allele vs. G-allele), (**B**) recessive (CC vs. GC + GG), (**C)** dominant (GG vs. GC + CC), (**D**) homozygous (CC vs. GG) and (**E)** heterozygous (GC vs. GG).

The meta-analysis of a possible association between the -137 polymorphism and risk of HCC is summarized in Tables 1 and 3. Based on the total study population, none of the five genetic models indicated a significant association: allelic model, OR 0.99, 95% CI 0.74–1.34, *P* = 0.97 (Fig. 2A); recessive model, OR 0.98, 95% CI 0.65–1.46, *P* = 0.91 (Fig. 2B); dominant model, OR 1.35, 95% CI 0.73–2.52, *P* = 0.34 (Fig. 2C); homozygous model, OR 0.99, 95% CI 0.65–1.49, *P* = 0.95 (Fig. 2D); heterozygous model, OR 0.99, 95% CI 0.66–1.48, *P* = 0.94 (Fig. 2E).

Next we meta-analyzed data for subgroups based on ethnicity or based on whether the HCC was related to chronic HBV infection. Meta-analysis of 7 studies^12^,^13^,^14^,^16^,^17^,^18^,^19^ involving 1,206 Asian cases and 2,052 Asian controls showed no evidence of a significant association between the -137 polymorphism and HCC risk for any of the five genetic models (Table 3): allelic, OR 1.01, 95% CI 0.71–1.43, *P* = 0.97; recessive model, OR 1.08, 95% CI 0.68–1.70, *P* = 0.75; dominant, OR 0.99, 95% CI 0.62–1.57, *P* = 0.96; homozygous, OR 1.09, 95% CI 0.69–1.72, *P* = 0.73; heterozygous, OR 0.98, 95% CI 0.61–1.56, *P* = 0.92. Similarly, no evidence of an association was identified in meta-analysis of 4 studies^14^,^17^,^18^,^19^ involving 832 Chinese cases and 1,151 Chinese controls (Table 3): allelic, OR 0.87, 95% CI 0.54–1.39, *P* = 0.56; recessive, OR 0.89, 95% CI 0.49–1.65, *P* = 0.72; dominant, OR 0.86, 95% CI 0.45–1.66, *P* = 0.66; homozygous, OR 0.80, 95% CI 0.43–1.48, *P* = 0.48; heterozygous, OR 0.85, 95% CI 0.44–1.66, *P* = 0.64.

Lastly, we meta-analyzed the subgroup of 978 cases and 1,752 controls in 6 studies^12^,^13^,^16^,^17^,^18^,^19^ in which the numbers of cases with HBV-related HCC were reported. The other two studies^14^,^15^ did not report results based on HCC etiology. Among the 6 studies, no evidence of an association between the -137 polymorphism and HBV-related HCC risk was observed (Table 3): allelic, OR 1.09, 95% CI 0.75–1.58, *P* = 0.65; recessive, OR 1.07, 95% CI 0.62–1.83, *P* = 0.82; dominant, OR 1.12, 95% CI 0.71–1.77, *P* = 0.63; homozygous, OR 1.17, 95% CI 0.68–2.01, *P* = 0.58; heterozygous, OR 1.11, 95% CI 0.69–1.76, *P* = 0.67.

### Meta-analysis of data on the -607 (rs1946518) polymorphism

**Figure 3:**
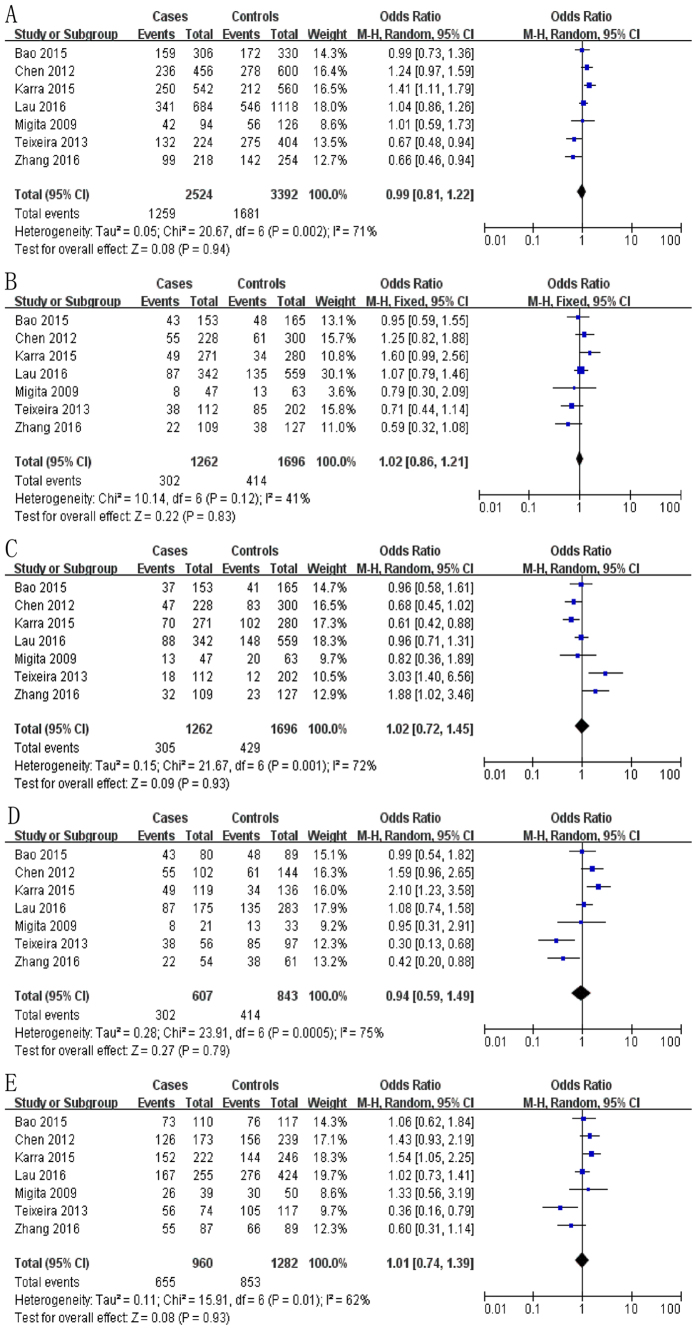
Forest plot describing the association between the -607 polymorphism (rs1946518) and risk of hepatocellular carcinoma across all study participants according to different genetic models. (**A**) allelic (C-allele vs. A-allele), (**B**) recessive (CC vs. AC + AA), (**C)** dominant (AA vs. AC + CC), (**D**) homozygous (CC vs. AA) and (**E)** heterozygous (AC vs. AA).

The meta-analysis of a possible association between the -607 polymorphism and risk of HCC is summarized in Tables 1 and 4. Based on the total study population^12^,^14^,^15^,^16^,^17^,^18^,^19^, none of the five genetic models indicated a significant risk: allelic, OR 0.99, 95% CI 0.81–1.22, *P* = 0.94 (Fig. 3A); recessive, OR 1.02, 95% CI 0.86–1.21, *P* = 0.83 (Fig. 3B); dominant, OR 1.02, 95% CI 0.72–1.45, *P* = 0.93 (Fig. 3C); homozygous, OR 0.94, 95% CI 0.59–1.49, *P* = 0.79 (Fig. 3D); heterozygous, OR 1.01, 95% CI 0.74–1.39, *P* = 0.93 (Fig. 3E).

Similarly, no significant association was observed for the subgroup of 1,150 Asian cases and 1,494 Asian controls in 6 studies^13^,^14^,^16^,^17^,^18^,^19^ (Table 4): allelic, OR 1.06, 95% CI 0.87–1.28, *P* = 0.55; recessive, OR 1.08, 95% CI 0.90–1.30, *P* = 0.43; dominant, OR 0.88, 95% CI 0.66–1.18, *P* = 0.39; homozygous, OR 1.12, 95% CI 0.75–1.68, *P* = 0.57; heterozygous, OR 1.17, 95% CI 0.97–1.40, *P* = 0.10. The same lack of association was observed for the subgroup of 832 Chinese cases and 1,151 Chinese controls in 4 studies^14^,^17^,^18^,^19^ (Table 4): allelic, OR 0.99, 95% CI 0.79–1.23, *P* = 0.92; recessive, OR 1.01, 95% CI 0.82–1.25, *P* = 0.90; dominant, OR 0.99, 95% CI 0.70–1.40, *P* = 0.96; homozygous, OR 0.98, 95% CI 0.62–1.54, *P* = 0.93; heterozygous, OR 1.05, 95% CI 0.85–1.31, *P* = 0.64.

Lastly, we meta-analyzed the subgroup of 922 cases and 1194 controls in 5 studies^12^,^16^,^17^,^18^,^19^ in which the numbers of cases with HBV-related HCC was reported. No evidence of an association between the -607 polymorphism and HBV-related HCC risk was observed (Table 4): allelic, OR 1.02, 95% CI 0.800–1.28, *P* = 0.89; recessive, OR 1.04, 95% CI 0.84–1.28, *P* = 0.71; dominant, OR 0.94, 95% CI 0.67–1.33, *P* = 0.73; homozygous, OR 1.03, 95% CI 0.63–1.66, *P* = 0.92; heterozygous, OR 1.09, 95% CI 0.81–1.46, *P* = 0.57.

### Sensitivity analysis

The robustness of the meta-analysis of 8 studies examining a possible association between the -137 polymorphism and HCC risk was assessed by repeating the meta-analysis after excluding a study^13^ in which all control cases were chronically infected with HBV and the *P* value associated with HWE was less than 0.05. Deleting these data from the meta-analysis did not alter the results obtained using any of the five genetic models, whether for the entire study population, the Asian population, or patients with HBV-related HCC. (This study did not involve Chinese patients, so no subgroup analysis of Chinese participants was performed.)

The robustness of the meta-analysis of studies examining a possible association between the -607 polymorphism and HCC risk was assessed by repeating the meta-analysis after excluding a study^15^ in which the *P* value associated with HWE was less than 0.05. Deleting these data from the meta-analysis did not alter the results obtained using any of the five genetic models across the entire study population as well as in the three subgroups.

### Publication bias

**Figure 4:**
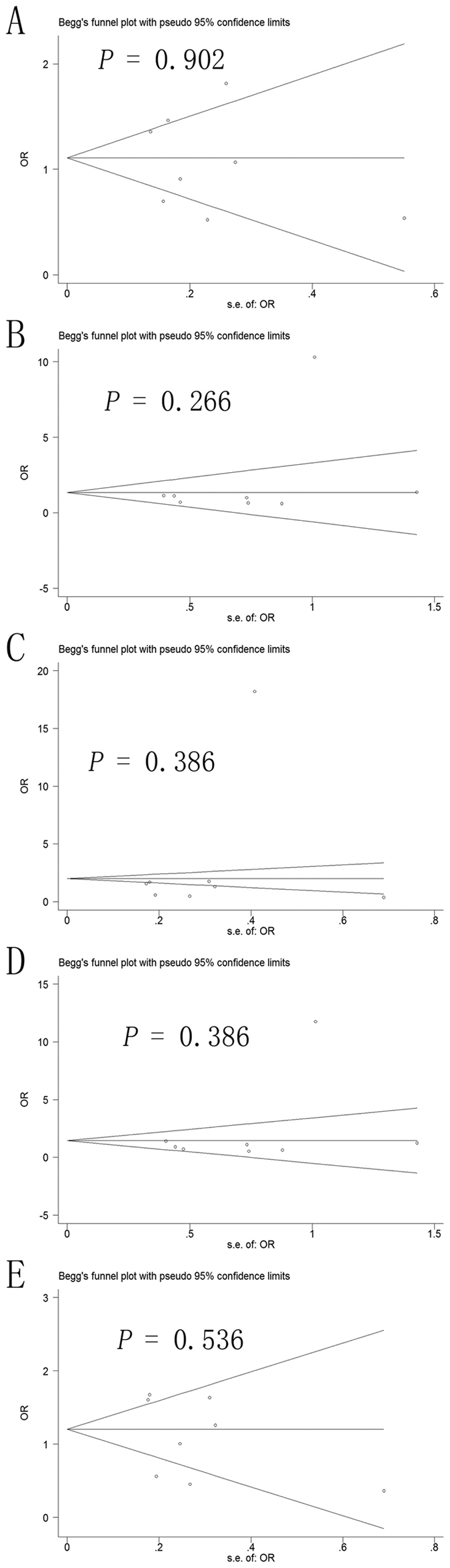
Begg’s funnel plot to assess publication bias in the meta-analysis of a potential association between the -137 polymorphism (rs187238) and risk of hepatocellular carcinoma across all study participants according to different genetic models. (**A**) allelic (C-allele vs. G-allele), (**B**) recessive (CC vs. GC + GG), (**C)** dominant (GG vs. GC + CC), (**D**) homozygous (CC vs. GG) and (**E)** heterozygous (GC vs. GG).

**Figure 5:**
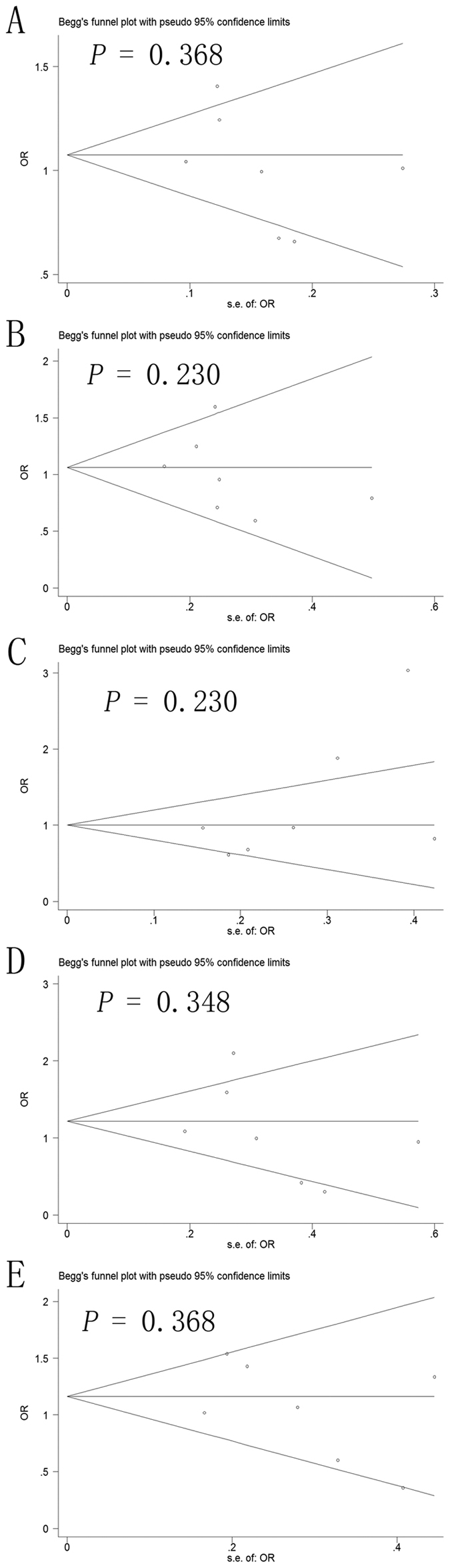
Begg’s funnel plot to assess publication bias in the meta-analysis of a potential association between the -607 polymorphism (rs1946518) and risk of hepatocellular carcinoma across all study participants according to different genetic models. (**A**) allelic (C-allele vs. A-allele), (**B**) recessive (CC vs. AC + AA), (**C)** dominant (AA vs. AC + CC), (**D**) homozygous (CC vs. AA) and (**E**) heterozygous (AC vs. AA).

Potential publication bias in this meta-analysis was assessed using Begg’s funnel plot and Egger’s test. No obvious asymmetry was observed in Begg’s funnel plots of the allelic modeling of the -137 polymorphism (Fig. 4) or -607 polymorphism (Fig. 5). *P* values for Egger’s tests were greater than 0.05 for the -137 polymorphism results based on any of the genetic models: allelic, *P* = 0.678; recessive, *P* = 0.307; dominant, *P* = 0.318; homozygous, *P* = 0.327; heterozygous, *P* = 0.454. Similarly, *P* values were greater than 0.05 for the -607 polymorphism results: allelic, *P* = 0.293; recessive, *P* = 0.476; dominant, *P* = 0.136; homozygous, *P* = 0.400; heterozygous, *P* = 0.328. These results suggest no potential publication bias.

## Discussion

Here we evaluate available evidence in English and Chinese on the possible association of polymorphisms -137 and -607 within the IL-18 promoter with risk of HCC^12^,^13^,^14^,^15^,^16^,^17^,^18^,^19^. We found no strong evidence of association, regardless of whether patients were Asian or not, and regardless of whether their HCC was HBV-related or not. Our results contrast with those of 5 case-control studies that reported an association between the -137 polymorphism and HCC risk^13^,^14^,^16^,^17^,^18^,^19^.

While our meta-analysis suggests that the -137 polymorphism is not helpful for predicting HCC risk, it may be useful for predicting metastasis or recurrence. Teixeira *et al*.^15^ found a significant association between this polymorphism and the presence of metastasis, while Chen *et al*.^14^ showed that a GC/CC genotype at the -137 position was significantly associated with recurrence. Future large studies should investigate this possibility.

We found no evidence of association between the -607 polymorphism and HCC risk for any patient subgroup examined. This contrasts with a large meta-analysis^40^ involving 3,117 controls and 2,137 cases with various types of cancers except HCC; that meta-analysis concluded that the -607 polymorphism increases risk of cancer in Asians but not in European Caucasians or Africans. Our results suggest that further research on the -607 polymorphism should focus on cancers other than HCC.

Our findings cast doubt on the relevance of these IL-18-related polymorphisms in the pathogenesis of HCC in general, and specifically HCC related to chronic HBV or HCV infection. These polymorphisms may not be appropriate genetic markers of HCC risk in individuals chronically infected with these viruses, so future research may wish to focus on other candidate markers^2^,^41^,^42^,^43^. It remains possible that other polymorphisms affecting IL-18 expression and activity influence HCC risk, perhaps because IL-18 plays a significant role in inflammation and immune responses, which may affect hepatitis outcomes, and/or because IL-18 can help clear HBV and HCV^33^,^34^,^35^. It may be that IL-18-related polymorphisms that promote viral clearance can influence the development of HCC. If so, then our results suggest that the -137 and -607 polymorphisms do not affect viral clearance, at least not clearance of HBV, since we found no evidence that either association is associated with risk of HBV-related HCC. Unfortunately few studies have examined HCC patients chronically infected with HCV, so further work is needed to explore whether these polymorphisms may affect HCV clearance.

To the best of our knowledge, this is the first meta-analysis evaluating the potential association of these two IL-18-related polymorphisms and susceptibility to HCC. At the same time, the work has several limitations that may affect interpretation of the results. First, the *P* value for HWE in one study^13^ on the -137 polymorphism was less than 0.05, which may be because all controls were chronically infected with HBV. The *P* value for HWE in one study^15^ on the -607 polymorphism was also less than 0.05. These results suggest that these study populations may not be representative of the broader target population. Nevertheless, sensitivity analyses showed that deleting these studies from the respective meta-analysis did not alter the results. Second, our exclusion of unpublished data and of papers published in languages other than English and Chinese may have biased our results. Third, the studies in our meta-analysis relied on an array of genotyping methods, including LM-PCR, Taqman, PCR-SSP, PCR-RFLP and PCR-LDR. Fourth, the studies may be subject to performance bias, attrition bias and reporting bias, although Newcastle-Ottawa scores were more than 5 for all 8 studies, indicating high quality. Fifth, the results may be affected by additional confounding factors, such as tumor status, gender and age, and we could not take this into account in the meta-analyses because studies either did not report these baseline data or they aggregated the data in different ways. Lastly, since this meta-analysis included few studies from non-Asian populations, large and well-designed studies in Caucasian and African populations are warranted. In particular, studies enrolling patients with HCV-related cases are needed since HCC pathogenesis differs depending on whether the patient is chronically infected with HBV or HCV.

In conclusion, this meta-analysis suggests that the IL-18-related -137 and -607 polymorphisms may not be associated with HCC risk, especially HBV-related HCC risk. Further detailed investigations involving larger, multi-ethnic samples are needed in order to clarify the role of these polymorphisms in HCC risk. Such work should also aim to explore gene-gene and gene-environment interactions that may mediate the association of -137 and -607 polymorphisms with development of HCC.

## Materials and Methods

### Search strategy

PubMed, EMBASE, Google Scholar and the Chinese National Knowledge Infrastructure (CNKI) databases were systematically searched for clinical and experimental case–control studies of associations of the -137 polymorphism (rs187238) and/or the -607 polymorphism (rs1946518) in the IL-18 promoter with HCC risk published through September 23, 2016 in English or Chinese. The following search strings were used: *interleukin-18* -*137*; *interleukin-18* -*607*; *IL-18* -*137; IL-18* -*607; rs187238; rs1946518;* these six terms in combination with *polymorphism, polymorphisms, SNP, variant, variants, variation, genotype, genetic* or *mutation;* and all of the above terms in combination with *hepatocellular carcinoma* or *liver cancer*. Reference lists in identified articles and reviews were also searched manually to identify additional eligible studies.

### Inclusion criteria

To be included in our review and meta-analysis, studies had to satisfy the following criteria: (1) a case-control design was used to assess the association of the rs187238 and/or rs1946518 polymorphisms with HCC risk in humans; (2) full text was available, and sufficient data were reported to estimate an odds ratio (OR) with 95% confidence interval (CI); and (3) genotype frequencies were reported. If multiple publications from the same research group appeared to report data for the same cases and controls, we included only the most recent publication in our meta-analysis.

### Data extraction

Two authors (SLZ and YZ) independently extracted the following data from included studies: first author’s family name, year of publication, ethnicity, country of origin, testing methods, *P* value for HWE in controls, numbers and genotypes of cases and controls, frequencies of genotypes in cases and controls. Discrepancies were resolved by consensus.

### Assessment of methodological quality

To assess the quality of the studies included in this analysis, the Newcastle–Ottawa Scale^44^ was used by two independent assessors (SLZ and XYH). For the Newcastle–Ottawa Scale, a maximum of nine points can be allotted to a study, with higher scores indicating better quality. Differences in quality score outcomes between the two assessors were solved by consensus. If consensus was not reached, a third assessor (LQL) was consulted for the final decision. The mean score for the included studies was 6.25 (Table 2).

### Statistical analysis

The unadjusted OR with 95% CI was used as described^45^,^46^,^47^ to assess the strength of the association of the rs187238 and rs1946518 polymorphisms with HCC risk based on the genotype frequencies in cases and controls. The significance of pooled ORs was determined using the Z test, with *P* < 0.05 defined as the significance threshold. Meta-analysis was conducted using a fixed-effect model when *P* > 0.10 for the Q test, indicating lack of heterogeneity among studies; otherwise, a random-effect model was used. All statistical tests for meta-analyses were performed using Review Manager 5.2 (Cochrane Collaboration). Publication bias was assessed using Begg’s funnel plot and Egger’s weighted regression in Stata 12.0 (Stata Corp, College Station, TX, USA), with *P* < 0.05 considered statistically significant.

## Additional Information

**How to cite this article:** Zhu, S.-L. *et al*. Genetic polymorphisms -137 (rs187238) and -607 (rs1946518) in the interleukin-18 promoter may not be associated with development of hepatocellular carcinoma. *Sci. Rep.* **6**, 39404; doi: 10.1038/srep39404 (2016).

**Publisher's note:** Springer Nature remains neutral with regard to jurisdictional claims in published maps and institutional affiliations.

## Figures and Tables

**Table 1:** Characteristics of studies in the meta-analysis.

First author	Year	Ethnicity	Country	Genotyping method	Type of control	*P* for HWE	Cases/Controls	No. of cases			Allele frequencies of cases, n, (%)		No. of controls			Allele frequencies of controls, n, (%)	
**IL-18 -137 (rs187238)**								GG	GC	CC	G	C	GG	GC	CC	G	C
Migita^12^	2009	Asian	Japan	LM-PCR	Healthy	0.531	47/63	43	3	1	89 (94.7)	5 (5.3)	52	10	1	114 (90.5)	12 (9.5)
Kim^13^	2009	Asian	South Korea	Taqman	CHB	0.031	56/558	37	17	2	91 (81.3)	21 (18.7)	434	122	2	990 (88.7)	126 (11.3)
Chen^14^	2012	Asian	China	PCR-SSP	Healthy	0.183	228/300	159	59	10	377 (82.7)	79 (17.3)	173	115	12	461 (76.8)	139 (23.2)
Teixeira^15^	2013	Mixed	Brazil	PCR-SSP	Healthy	0.952	112/202	57	48	7	162 (72.3)	62 (27.7)	100	84	18	284 (70.3)	120 (29.7)
Karra^16^	2015	Asian	India	PCR-SSP	Healthy	0.320	271/280	123	134	14	380 (70.2)	162 (29.8)	159	108	13	426 (76.0)	134 (24.0)
Bao^17^	2015	Asian	China	PCR-RFLP	Healthy	0.548	153/165	122	28	3	272 (88.9)	34 (11.1)	106	54	5	266 (80.6)	64 (19.4)
Zhang^18^	2016	Asian	China	PCR-LDR	Healthy	0.110	109/127	82	25	2	189 (86.7)	29 (13.3)	99	24	4	222 (87.4)	32 (12.6)
Lau^19^	2016	Asian	Taiwan, China	Taqman	Healthy	0.370	342/559	266	73	3	605 (88.5)	79 (11.5)	476	78	5	1030 (92.1)	88 (7.9)
**IL18 -607 (rs1946518)**								AA	AC	CC	A	C	AA	AC	CC	A	C
Migita^12^	2009	Asian	Japan	LM-PCR	Healthy	0.777	47/63	13	26	8	52 (55.3)	42 (44.7)	20	30	13	70 (55.6)	56 (44.4)
Chen^14^	2012	Asian	China	PCR-SSP	Healthy	0.429	228/300	47	126	55	220 (48.2)	236 (51.8)	83	156	61	322 (53.7)	278 (46.3)
Teixeira^15^	2013	Mixed	Brazil	PCR-SSP	Healthy	0.005	112/202	18	56	38	92 (41.1)	132 (58.9)	12	105	85	129 (31.9)	275 (68.1)
Karra^16^	2015	Asian	India	PCR-SSP	Healthy	0.120	271/280	70	152	49	292 (53.8)	250 (46.2)	102	144	34	348 (62.1)	212 (37.8)
Bao^17^	2015	Asian	China	PCR-RFLP	Healthy	0.322	153/165	37	73	43	147 (48.0)	159 (52.0)	41	76	48	158 (47.9)	172 (52.1)
Zhang^18^	2016	Asian	China	PCR-LDR	Healthy	0.542	109/127	32	55	22	119 (54.6)	99 (45.4)	23	66	38	112 (44.1)	142 (55.9)
Lau^19^	2016	Asian	Taiwan, China	Taqman	Healthy	0.777	342/559	88	167	87	343 (50.1)	341 (49.9)	148	276	135	572 (51.2)	546 (48.8)

IL18, interleukin-18; LM, ligation-mediated; PCR, polymerase chain reaction; SSP, sequence-specific primer; RFLP, restriction fragment length polymorphism; LDR, ligase detection reaction; CHB, chronically infected with hepatitis B virus; HWE, Hardy-Weinberg equilibrium.

**Table 2:** Methodological quality of case-control studies in our meta-analyses, based on the Newcastle–Ottawa Scale.

Study	Selection (score)				Comparability (score)	Exposure (score)			
	Adequate definition of patient cases	Representativeness of patients/cases	Selection of controls	Definition of controls	Control for important factor or additional factor	Ascertainment of exposure (blinding)	Same method of ascertainment for participants	Non-response rate^a^	Total score^b^
Migita *et al*.^12^	1	1	0	1	0	0	1	1	5
Kim *et al*.^13^	1	1	0	1	0	0	1	1	5
Chen *et al*.^14^	1	1	1	1	1	0	1	1	7
Teixeira *et al*.^15^	1	1	1	1	0	0	1	1	6
Karra *et al*.^16^	1	1	0	1	0	0	1	1	5
Bao *et al*.^17^	1	1	1	1	2	0	1	1	8
Zhang *et al*.^18^	1	1	1	1	2	0	1	1	8
Lau *et al*.^19^	1	1	0	1	1	0	1	1	6

^a^One point was awarded when there was no significant difference in the response rate between both groups based on the chi-squared test (*P* > 0.05).

^b^Calculated by adding up the points awarded for each item.

**Table 3:** Overall meta-analysis of the association between the -137 polymorphism (rs187238) and risk of hepatocellular carcinoma.

Genotype comparison and genetic model	OR [95% CI]	Z (*P* value)	Heterogeneity of study design			Analysis model
			χ2	df (*P* value)	I^2^ (%)	
**IL-18 -137 (rs187238)) in total population from 8 case control studies (1,318 cases and 2,254 controls)**						
Allelic (C-allele vs. G-allele)	0.99 [0.74, 1.34]	0.03 (0.97)	30.47	7 (<0.001)	77	Random
Recessive (CC vs. GC + GG)	0.98 [0.65, 1.46]	0.12 (0.91)	6.98	7 (0.43)	0	Fixed
Dominant (GG vs. GC + CC)	1.35 [0.73, 2.52]	0.96 (0.34)	83.15	7 (<0.001)	92	Random
Homozygous (CC vs. GG)	0.99 [0.65, 1.49]	0.06 (0.95)	8.41	7 (0.30)	17	Fixed
Heterozygous (GC vs. GG)	0.99 [0.66, 1.48]	0.07 (0.94)	37.78	7 (<0.001)	81	Random
**IL-18 -137 (rs187238) in Asian population from 7 case-control studies (1,206 cases and 2,052 controls)**						
Allelic (C-allele vs. G-allele)	1.01 [0.71, 1.43]	0.03 (0.97)	29.85	6 (<0.001)	80	Random
Recessive (CC vs. GC + GG)	1.08 [0.68, 1.70]	0.31 (0.75)	6.06	6 (0.42)	1	Fixed
Dominant (GG vs. GC + CC)	0.99 [0.62, 1.57]	0.05 (0.96)	37.39	6 (<0.001)	84	Random
Homozygous (CC vs. GG)	1.09 [0.69, 1.72]	0.35 (0.73)	7.48	6 (0.28)	20	Fixed
Heterozygous (GC vs. GG)	0.98 [0.61, 1.56]	0.10 (0.92)	37.70	6 (<0.001)	84	Random
**IL-18 -137 (rs187238) in Chinese population from 4 case-control studies (832 cases and 1,151 controls)**						
Allelic (C-allele vs. G-allele)	0.87 [0.54, 1.39]	0.58 (0.56)	17.54	3 (<0.001)	83	Random
Recessive (CC vs. GC + GG)	0.89 [0.49, 1.65]	0.36 (0.72)	0.70	3 (0.87)	0	Fixed
Dominant (GG vs. GC + CC)	0.86 [0.45, 1.66]	0.44 (0.66)	25.42	3 (<0.001)	88	Random
Homozygous (CC vs. GG)	0.80 [0.43, 1.48]	0.71 (0.48)	0.68	3 (0.88)	0	Fixed
Heterozygous (GC vs. GG)	0.85 [0.44, 1.66]	0.47 (0.64)	25.71	3 (<0.001)	88	Random
**IL-18 -137 (rs187238) in patients with hepatitis B virus-related HCC from 6 case-control studies (978 cases and 1,752 controls)**						
Allelic (C-allele vs. G-allele)	1.09 [0.75, 1.58]	0.45 (0.65)	20.51	5 (0.001)	76	Random
Recessive (CC vs. GC + GG)	1.07 [0.62, 1.83]	0.23 (0.82)	6.07	5 (0.30)	18	Fixed
Dominant (GG vs. GC + CC)	1.12 [0.71, 1.77]	0.48 (0.63)	22.56	5 (<0.001)	78	Random
Homozygous (CC vs. GG)	1.17 [0.68, 2.01]	0.56 (0.58)	7.12	5 (0.21)	30	Fixed
Heterozygous (GC vs. GG)	1.11 [0.69, 1.76]	0.42 (0.67)	23.04	5 (<0.001)	78	Random

HCC, hepatocellular carcinoma; IL18, interleukin-18; OR, odds ratio; 95% CI, 95% confidence interval.

**Table 4:** Overall meta-analysis of the association between the -607 polymorphism (rs1946518) and risk of hepatocellular carcinoma.

Genotype comparison and genetic model	OR [95% CI]	Z (*P* value)	Heterogeneity of study design			Analysis model
			χ2	df (*P* value)	I^2^ (%)	
**IL18 -607 (rs1946518) in total population from 7 case-control studies (1,262 cases and 1,696 controls)**						
Allelic (C-allele vs. A-allele)	0.99 [0.81, 1.22]	0.08 (0.94)	20.67	6 (0.002)	71	Random
Recessive (CC vs. AC + AA)	1.02 [0.86, 1.21]	0.22 (0.83)	10.14	6 (0.12)	41	Fixed
Dominant (AA vs. AC + CC)	1.02 [0.72, 1.45]	0.09 (0.93)	21.67	6 (0.001)	72	Random
Homozygous (CC vs. AA)	0.94 [0.59, 1.49]	0.27 (0.79)	23.91	6 (<0.001)	75	Random
Heterozygous (AC vs. AA)	1.01 [0.74, 1.39]	0.08 (0.93)	15.91	6 (0.01)	62	Random
**IL18 -607 (rs1946518) in Asian population from 6 case-control studies (1,150 cases and 1,494 controls)**						
Allelic (C-allele vs. A-allele)	1.06 [0.87, 1.28]	0.59 (0.55)	13.51	5 (0.02)	63	Random
Recessive (CC vs. AC + AA)	1.08 [0.90, 1.30]	0.79 (0.43)	7.55	5 (0.18)	34	Fixed
Dominant (AA vs. AC + CC)	0.88 [0.66, 1.18]	0.86 (0.39)	11.74	5 (0.04)	57	Random
Homozygous (CC vs. AA)	1.12 [0.75, 1.68]	0.56 (0.57)	13.89	5 (0.02)	64	Random
Heterozygous (AC vs. AA)	1.17 [0.97, 1.40]	1.62 (0.10)	7.86	5 (0.16)	36	Fixed
**IL18 -607 (rs1946518) in Chinese population from 4 case-control studies (832 cases and 1,151 controls)**						
Allelic (C-allele vs. A-allele)	0.99 [0.79, 1.23]	0.10 (0.92)	8.23	3 (0.04)	64	Random
Recessive (CC vs. AC + AA)	1.01 [0.82, 1.25]	0.12 (0.90)	4.19	3 (0.24)	28	Fixed
Dominant (AA vs. AC + CC)	0.99 [0.70, 1.40]	0.05 (0.96)	7.39	3 (0.06)	59	Random
Homozygous (CC vs. AA)	0.98 [0.62, 1.54]	0.09 (0.93)	8.46	3 (0.04)	65	Random
Heterozygous (AC vs. AA)	1.05 [0.85, 1.31]	0.47 (0.64)	4.92	3 (0.18)	39	Fixed
**IL18 -607 (rs1946518) in patients with hepatitis B virus-related HCC from 5 case-control studies (922 cases and 1194 controls)**						
Allelic (C-allele vs. A-allele)	1.02 [0.80, 1.28]	0.13 (0.89)	12.20	4 (0.02)	67	Random
Recessive (CC vs. AC + AA)	1.04 [0.84, 1.28]	0.37 (0.71)	6.97	4 (0.14)	43	Fixed
Dominant (AA vs. AC + CC)	0.94 [0.67, 1.33]	0.34 (0.73)	10.31	4 (0.04)	61	Random
Homozygous (CC vs. AA)	1.03 [0.63, 1.66]	0.10 (0.92)	12.29	4 (0.02)	67	Random
Heterozygous (AC vs. AA)	1.09 [0.81, 1.46]	0.57 (0.57)	6.81	4 (0.15)	41	Fixed

HCC, hepatocellular carcinoma; IL18, interleukin-18; OR, odds ratio; 95% CI, 95% confidence interval.
